# Magnoflorine Alleviates “M1” Polarized Macrophage-Induced Intervertebral Disc Degeneration Through Repressing the HMGB1/Myd88/NF-κB Pathway and NLRP3 Inflammasome

**DOI:** 10.3389/fphar.2021.701087

**Published:** 2021-07-23

**Authors:** Feng Zhao, Zhenye Guo, Fushan Hou, Wei Fan, Binqiang Wu, Zhonglai Qian

**Affiliations:** ^1^ Department of Orthopedics, The First Affiliated Hospital of Soochow University, Suzhou 215006, China; ^2^ Department of Orthopedics, The Second Hospital of Shanxi Medical University, Taiyuan 030001, China

**Keywords:** magnoflorine, macrophages, nucleus pulposus cells, intervertebral disc degeneration, inflammatory response, NLRP3

## Abstract

Intervertebral disc degeneration (IDD) is related to the deterioration of nucleus pulposus (NP) cells due to hypertrophic differentiation and calcification. The imbalance of pro-inflammatory (M1 type) and anti-inflammatory (M2 type) macrophages contributes to maintaining tissue integrity. Here, we aimed to probe the effect of Magnoflorine (MAG) on NP cell apoptosis mediated by “M1” polarized macrophages. THP-1 cells were treated with lipopolysaccharide (LPS) to induce “M1” polarized macrophages. Under the treatment with increasing concentrations of MAG, the expression of pro-inflammatory cytokines (IL-1β, IL-6, TNF-α, IL-18), high mobility group box protein 1 (HMGB1), as well as myeloid differentiation factor 88 (MyD88), nuclear factor kappa B (NF-κB) and NOD-like receptor 3 (NLRP3) inflammasomes in THP-1 cells were determined. What’s more, human NP cells were treated with the conditioned medium (CM) from THP-1 cells. The NP cell viability and apoptosis were evaluated. Western blot (WB) was adopted to monitor the expression of apoptosis-related proteins (Bax, Caspase3, and Caspase9), catabolic enzymes (MMP-3, MMP-13, ADAMTS-4, and ADAMTS-5), and extracellular matrix (ECM) compositions (collagen II and aggrecan) in NP cells. As a result, LPS evidently promoted the expression of pro-inflammatory cytokines and HMGB1, the MyD88-NF-κB activation, and the NLRP3 inflammasome profile in THP-1 cells, while MAG obviously inhibited the "M1″ polarization of THP-1 cells. After treatment with “M1” polarized THP-1 cell CM, NP cell viability was decreased, while cell apoptosis, the pro-inflammatory cytokines, apoptosis-related proteins, and catabolic enzymes were distinctly up-regulated, and ECM compositions were reduced. After treatment with MAG, NP cell damages were dramatically eased. Furthermore, MAG dampened the HMGB1 expression and inactivated the MyD88/NF-κB pathway and NLRP3 inflammasome in NP cells. In conclusion, this study confirmed that MAG alleviates “M1” polarized macrophage-mediated NP cell damage by inactivating the HMGB1-MyD88-NF-κB pathway and NLRP3 inflammasome, which provides a new reference for IDD treatment.

## Introduction

The intervertebral disc consists of the nucleus pulposus (NP), annulus fibrosus, and cartilaginous endplate, which is located in and attached to the conus (Raj, 2008). Intervertebral disc degeneration (IDD) is a major cause of back, neck, and nerve root pain, and it is related to changes in the structure, cell number, and composition of the intervertebral disc (Risbud and Shapiro, 2014; Kos et al., 2019). With the acceleration of population aging, the incidence of IDD increases year by year, which not only affects people's quality of life but also causes a certain economic burden (Vergroesen et al., 2015). Current IDD treatments mainly focus on reducing symptoms and cannot reverse the process of IDD, which leads to a high recurrence rate (Jiang and Lu, 2016). Therefore, it is necessary to develop new therapies for IDD.

Magnoflorine (MAG) is a quaternary aporphine alkaloid widely distributed within the representatives of several botanical families like Berberidaceae, Magnoliaceae, Papaveraceae, or Menispermaceae (Okon et al., 2020). Over the years, multiple studies have manifested that MAG has various pharmacological activities, such as anti-inflammatory, anti-oxidant, and immunoregulatory activities (Xu et al., 2020). Hence, MAG has received increasing attention and researches. For instance, MAG has been confirmed to abate inflammatory response in lipopolysaccharide (LPS)-induced acute lung injury in mice, possibly through inhibition of the Toll like receptor 4-mediated nuclear factor kappa B (NF-κB) and mitogen-activated protein kinase (MAPK) pathways (Guo et al., 2018). Furthermore, it has been revealed that MAG has therapeutic effects on peri-implant osteolysis caused by worn debris and diseases caused by chronic inflammation and the overactivation of osteoclasts (Sun et al., 2020). These findings demonstrate the medicinal value of MAG. Nevertheless, the function and potential mechanism of MAG in IDD remain to be further investigated.

As a vital member of the High Mobility Group-box superfamily, High-mobility group box 1 (HMGB1) functions as a nuclear DNA-binding protein and regulates transcription, and is involved in organization of DNA. Notably, multiple studies have revealed that HMGB1 plays a role in several cellular processes, including inflammation, cell differentiation and tumor cell migration (Andersson et al., 2018; Yang et al., 2020). For instance, it has been shown that the inflammatory response induced by periodontal infection stimulates the secretion of HMGB1, while inhibition of HMGB1 attenuates the levels of inflammatory cytokines in the mouse periodontitis model (Yoshihara-Hirata et al., 2018). Other reports have claimed that HMGB1 is highly expressed in osteoarthritis (OA) and is targeted by miR-140-5p. Further researches revealed that miR-140-5p reduces the expression of IL-1β-induced inflammatory cytokines, matrix metalloprotease and cell apoptosis by down-regulating HMGB1 in chondrocytes (Wang et al., 2020). Notably, several reports have confirmed that HMGB1 is overexpressed in IDD, and high levels of HMGB1 suppress NP cell proliferation, induce cell apoptosis and promote the degradation of the extracellular matrix (ECM), thus leading to IDD (Fu et al., 2020). Nevertheless, it is unclear whether MAG influences IDD progression through repressing HMGB1.

Myeloid differentiation primary response 88 (MyD88) belongs to an adaptor molecule of Toll-like receptor (TLR) signaling that initiates innate immunity by mediating inflammatory cytokines expression through the intracellular inflammatory signaling cascades such as the Jun kinase and nuclear factor kappa B (NF-κB) pathways (Lawrence, 2009; Deguine and Barton, 2014; Ju et al., 2018). Following the upregulation of MyD88, NF-κB p65 phosphorylation is increased and translocases into the nuclear and eventually promotes the generation of inflammatory cytokines, such as interleukin-6 (IL-6) and tumor necrosis factor-alpha (TNF-α) (Jiang et al., 2020). On the other hand, the NACHT, LRR, and PYD domains-containing protein 3 (NLRP3) inflammasome is an oligomeric complex comprised of the NOD-like receptor NLRP3, the adaptor apoptosis-associated speck-like protein (ASC), and caspase-1. Under pathological conditions, extracellular HMGB1 is recognized by TLR4 and promotes NF-κB p65 phosphorylation and the formation of NLRP3 inflammasome, thus enhancing the releasing of IL-1β and IL-18 and enhancing apoptosis (Xie et al., 2021). Additionally, the NLRP3 inflammasome activation also facilitates HMGB1 production (Chi et al., 20152015). Nicotinamide phosphoribosyltransferase (NAMPT) knockdown attenuated matrix degradation induced by TNF-α in nucleus pulposus (NP) cells through repressing NLRP3 inflammasome activity and NF-κB signaling (Shang et al., 2020). However, whether MAG exerts protective effects in NP cells via targeting HMGB1, MyD88/NF‐κB signaling and NLRP3 inflammasome is less understood.

Macrophages are a heterogeneous group of cells found in most vertebrate tissues. They maintain homeostasis and regulate the immune system, serving as the body's first line of defense against external damage (Dos Anjos Cassado, 2017). As the surrounding microenvironment changes, resting macrophages (M0) exhibit two different phenotypes, namely polarized “M1” and “M2” phenotypes, and play different roles. “M1” polarized macrophages can promote the production of inflammatory cytokines and exert anti-bacterial and anti-tumor effects. In contrast, “M2” polarized macrophages have anti-inflammatory and immunosuppressive effects (Artyomov et al., 2016; Essandoh et al., 2016; Verdeguer and Aouadi, 2017). A previous study has demonstrated that chronic inflammation of IDD is related to increased “M1” macrophages as there are accumulated CCR7+ and CD163 + cells in unhealthy nucleus pulposus (NP), annulus fibrosus (AF), and end plate (EP) regions exhibiting structural irregularities and defects (Nakazawa et al., 2018). However, the interaction and mechanism between proinflammatory macrophages and human NP cells remain elusive.

THP-1 is a monocyte cell line with the ability to differentiate into macrophages or dendritic cells (Chanput et al., 2015). The stable growth of nucleus pulposus (NP) cells is one of the important conditions to maintain the function of intervertebral disc (Kang et al., 2019). Here, we adopted in-vitro experiments to understand the role of MAG on the interaction between “M1” polarized macrophage and NP cells. Our data suggested that MAG alleviates "M1″ macrophage-mediated NP cell damage via the HMGB1-MyD88-NF-κB pathway, indicating the potential role of MAG in treating IDD.

## Materials and Methods

### Cell Culture

THP-1 cells (Cat. No. TCHu 57) were provided by the Cell Center of the Chinese Academy of Sciences (Shanghai, China). Human Nucleus Pulposus Cells (NP cell, Cat.No. 4800) were purchased from ScienCell Research Laboratories (Carlsbad, CA, United States). The cells were cultured in the DMEM medium (Thermo Fisher HyClone, UT, United States) containing 5% FBS (Thermo Fisher Scientific, MA, United States) in an incubator at 37°C with 5% CO_2_. NP cells and THP-1 cells in the logarithmic growth phase were isolated and trypsinized using 0.25% trypsin (Thermo Fisher HyClone, UT, United States). The third generation of the cells was applied for subsequent experiments.

THP-1 cells were treated with LPS (100 ng/ml) (Chenevier-Gobeaux et al., 2016) to induce the activation of "M1″ polarized macrophages. They were then treated with different concentrations of MAG (12.5, 25, 50, and 100 μg/ml) (Guo et al., 2018) to observe the effect of MAG. NP cells were treated with MAG after the IL-1β treatment (20 ng/ml) (Jiang et al., 2019). The role of the HMGB1 pathway in the above rocess was further investigated using the HMGB1 inhibitor Ethyl pyruvate (EP) (10 mM) (Chavali et al., 2020).

### Quantitative Real-Time PCR

THP-1 and NP cells treated with different factors were collected, and the TRIzol reagent (Invitrogen, Carlsbad, CA, United States) was employed to isolate the total RNA. Then, the purity and concentration of total RNA were detected using ultraviolet spectrophotometer (UV-1600PC, Mapada). Next, 2 μg RNA in each group was reversely transcribed into cDNA with the ReverTra Ace qPCR RT Kit (TOYOBO, Osaka, Japan). Later, the specific primers of IL-1β, IL-6, TNF-α and IL-18 were used for PCR amplification with the SYBR Green method (SYBR GreenRealtime PCR Master Mix, Code No.QPK-201, QPK-201T, TOYOBO, Tokyo, Japan) using cDNA as a template. The internal reference of IL-1β, IL-6, TNF-α, and IL-18 was β-Actin, and their relative expression was calculated with the 2^−ΔΔCT^ method. The primer sequences were as follows:

### Enzyme-Linked Immunosorbent Assay

THP-1 and NP cells treated with LPS, or IL-1β or different doses of MAG were plated into 6-well plates, and three repetitive wells were set in each group. After 24 h of culture, the cells were centrifuged at 4°C for 10 min at 1000 RPM, and the supernatant was taken. The HMGB1 content in the supernatant was measured by the HMGB1 ELISA kit (Cat.No. ARG81351, Arigo Biolaboratories Corp., Taiwan) according to the manufacturer's instructions. The optical density (OD) value was read at 450 nm on a Microplate reader (Multiskan FC, Thermo Fisher HyClone, UT, United States).

### Western Blot

THP-1 and NP cells treated with different factors were taken. Then, they were treated with the RIPA lysis buffer (Beyotime Biotechnology, Shanghai, China) and collected through low-speed centrifugation. The nuclear and cytoplasmic Protein Extraction Kit (NO. C510001, Sangon Biotech, Shanghai, China) was used for extracting nuclear or cytoplasmic proteins according to the instructions of the manufacturer. Afterward, the total protein was extracted, and the protein concentration was determined by the Bradford method. The protein sample was boiled for 5 min, cooled on ice, and centrifuged for 30 s. The protein sample (20 μg) was applied for polyacrylamide gel electrophoresis and transferred to PVDF membranes (Millipore, Bedford, MA, United States) at 100 V for 1 h. The membranes were then blocked with 5% skim milk at room temperature (RT) for 1 h. Next, they were incubated at 4°C overnight with the following antibodies: Anti-MyD88 antibody (1:2,000, ab133739), Lamin B (1:500, ab32535), Anti-p-NF-κB p65 antibody (1:1,000, ab76302), Anti-NF-κB p65 antibody (1:2,000, ab16502), Anti-NLRP3 antibody (1:1,000, ab263899), Anti-ASC antibody (1:2,000, ab151700), Anti-Caspase1 antibody (1:1,000, ab27802), Anti-Bax antibody (1:1,000, ab32503), Anti-Caspase3 antibody (1:2000, ab184787), Anti-Caspase9 antibody (1:2,000, ab202068), Anti-MMP-3 antibody (1:2000, ab52915), Anti-MMP-13 antibody (1:4,000, ab39012), Anti-ADAMTS-4 antibody (1:1,000, ab185722), Anti-ADAMTS-5 antibody (1:250, ab41037), Anti-collagen II antibody (1:2,000, ab188570), Anti-aggrecan antibody (1:1,000, ab3778), and Anti-β-Actin antibody (1:1,000, ab8227). Subsequently, the membranes were rinsed twice with TBST and incubated with fluorescein-labeled anti-rabbit antibody (1:2,500, ab6721) or anti-mouse antibody (1:1,000, ab190475) at RT for 1 h. After being rinsed 3 times, the membranes were exposed with the ECL chromogenic agent (Beyotime Biotechnology, Shanghai, China), and a membrane scanner was applied for imaging. All the above antibodies were purchased from Abcam company (Shanghai, China).

### Cell Counting Kit-8 Assay

NP cells treated with IL-1β/MAG or conditional medium from THP1 cells were plated into 96-well plates (2×10^3^ cells/well). Then, 10 μL CCK-8 solution (MedChem Express, NJ, United States) was supplemented 24, 48, and 72 h after the culture, and the cells were further incubated for 4 h. The optical density (OD) at 450 nm was observed with a microplate reader (Multiskan Spectrum, Thermo Scientific Microplate Reader, Shanghai, China), and the cell growth curve was plotted to evaluate the proliferative ability of the cells.

### Flow Cytometry

NP cells treated with IL-1β/MAG or conditional medium from THP1 cells were digested with 0.25% trypsin and collected by centrifugation (at 1,000 RPM for 4 min). After the cells were rinsed with the buffer solution 3 times, they were resuspended with the binding buffer and adjusted to reach a density of 1×10^6^ cells/mL. Subsequently, FITC-Annexin V and PI solution (BD Biosciences, NJ, United States) were added and incubated in the dark for 15 min, and FCM was utilized to verify cell apoptosis on BD FACSCelesta™ Flow Cytometer (BD company).

### Cellular Immunofluorescence

THP-1 cells were seeded in 24-well plate (5×10^5^ cells per well). After being treated with LPS, MAG or EP, the cells were fixed with 4% paraformaldehyde (room temperature, 15 min). Next, the cells were washed with PBS for 3 times (5 min each time) and 5% goat serum was used for blocking the cells. Followed by that, the cells were incubated with primary antibodies, including p-NF-κB (1:150, ab76302, Abcam) and anti-NLRP3 (1:100, ab263899, Abcam) for 15 h at 4°C. Then the cells were washing 3 times with PBS, and incubated with the secondary antibodies, including the Cy3-conjugated goat anti-rabbit IgG (A0562, Beyotime, Shanghai, China) at room temperature for 1 h. Next, the nucleus of cells was stained by DAPI (1:4, Beyotime, Shanghai, China). Finally, the immunofluorescence signals were observed using an Olympus-IX71 fluorescent microscope (Olympus Corporation, Tokyo, Japan).

### Statistical Analysis

Prism 8.02 (GraphPad Software, United States) was applied to analyze the experimental results, and the measurement data was present as mean ± SD (x ± s). *t* test was adopted for comparison between two groups, and one-way ANOVA followed by Tukey’s post hoc test was employed for comparison between multiple groups. *p* < 0.05 was considered statistically significant.

## Results

### MAG Repressed the Inflammatory Response of "M1″ Polarized Macrophages THP1 Cells

**FIGURE 1:**
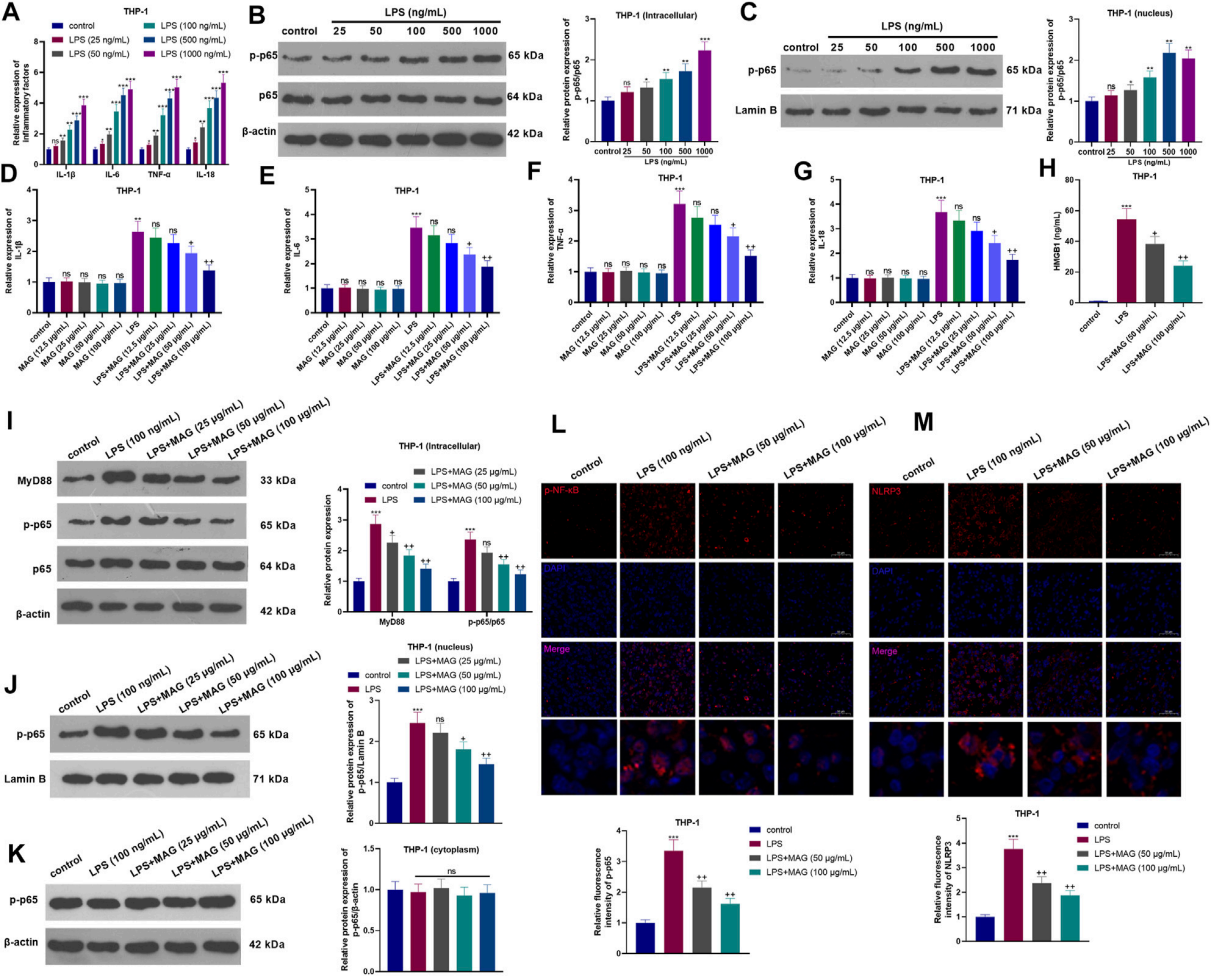
Effects of MAG on the inflammation of “M1” polarized macrophages. THP-1 cells were treated with varying doses of LPS (25 ng/ml, 50 ng/ml, 100 ng/ml, 500 ng/ml, and 1,000 ng/ml) for 24 h. **(A)**. The expression of IL-1β, IL-6, TNF-α, and IL-18 was compared by qRT-PCR. **(B-C)**. Western blot was conducted to detect NF-κB pathway in THP1 cells. After treatment without or with LPS (100 ng/ml) for 24 h, THP-1 cells were treated with varying concentrations of MAG (12.5, 25, 50, and 100 μg/ml) for 24 h. **(D-G)**. The expression of IL-1β, IL-6, TNF-α, and IL-18 was compared by qRT-PCR. **(H)**. ELISA was applied to detect HMGB1 level in the supernatant of THP1 cells. **(I-K)**. Western blot verified the expression of the MyD88-NF-κB pathway in THP1 cells. **(L-M)**. Cell immunofluorescence was performed to detect p-NF-κB and NLRP3 in the TBP1 cells (×200). *n* = 3. ANOVA *p* < 0.01. ns *p* > 0.05, **p < 0.01, ***p < 0.001 (vs control group). ns *p* > 0.05, +*p* < 0.05, ++*p* < 0.01 (vs LPS group).

After the treatment with different dose of LPS (25–1,000 ng/ml) for 24 h, the inflammatory cytokines and NF-κB pathway expressions in THP-1 cells were determined. As the data shown, LPS significantly promoted IL-1β, IL-6, TNF-α, and IL-18 mRNA expression, NF-κB phosphorylation and nuclear translocation of phosphorylated NF-κB (Figures 1A–C). Next, THP1 cells treated with different concentrations of MAG (12.5, 25, 50, and 100 μg/ml) were dealt with or without LPS (100 ng/ml) for 24 h. Next, the inflammatory cytokines (IL-1β, IL-6, TNF-α, and IL-18) in THP-1 cells were examined by RT-PCR. The results manifested that compared with the control group, MAG had no significant effect on the expression of inflammatory cytokines (*p* > 0.05), however, LPS increased the expression of inflammatory cytokines. In contrast, higher doses of MAG (50, 100 μg/ml) impeded the levels of inflammatory cytokines (vs LPS group) (*p* < 0.05, Figures 1D–G). ELISA results illustrated that the HMGB1 content in the culture medium of THP-1 cells was distinctly increased after LPS treatment, but the HMGB1 expression was inhibited by MAG compared with that of the LPS group (*p* < 0.05, Figure 1H). Furthermore, Western blot and immunofluorescence were conducted to evaluate MyD88-NF-κB pathway and NLRP3 inflammasome in THP1 cells. As the results showed, LPS facilitated the expression of MyD88 and p-NF-κB p65 in the whole cells (Figure 1I) as well as nucleus translocation of p-NF-κB p65 (Figure 1J), but not altered p-NF-κB p65 level in the cytoplasm (Figures 1K,L). In addition, LPS also enhanced the expression of NLRP3 inflammasome in THP1 cells (Figure 1M). However, the treatment of MAG signficantly reduced MyD88-NF-κB pathway activation and NLRP3 inflammasome in THP1 cells (vs the LPS group, *p* < 0.05, Figures 1I–M). These findings suggested that MAG had an inhibitory effect on LPS-induced “M1” polarized macrophage inflammation.

### Effects of MAG on IL-1β-Mediated NP Cell Damage

**FIGURE 2:**
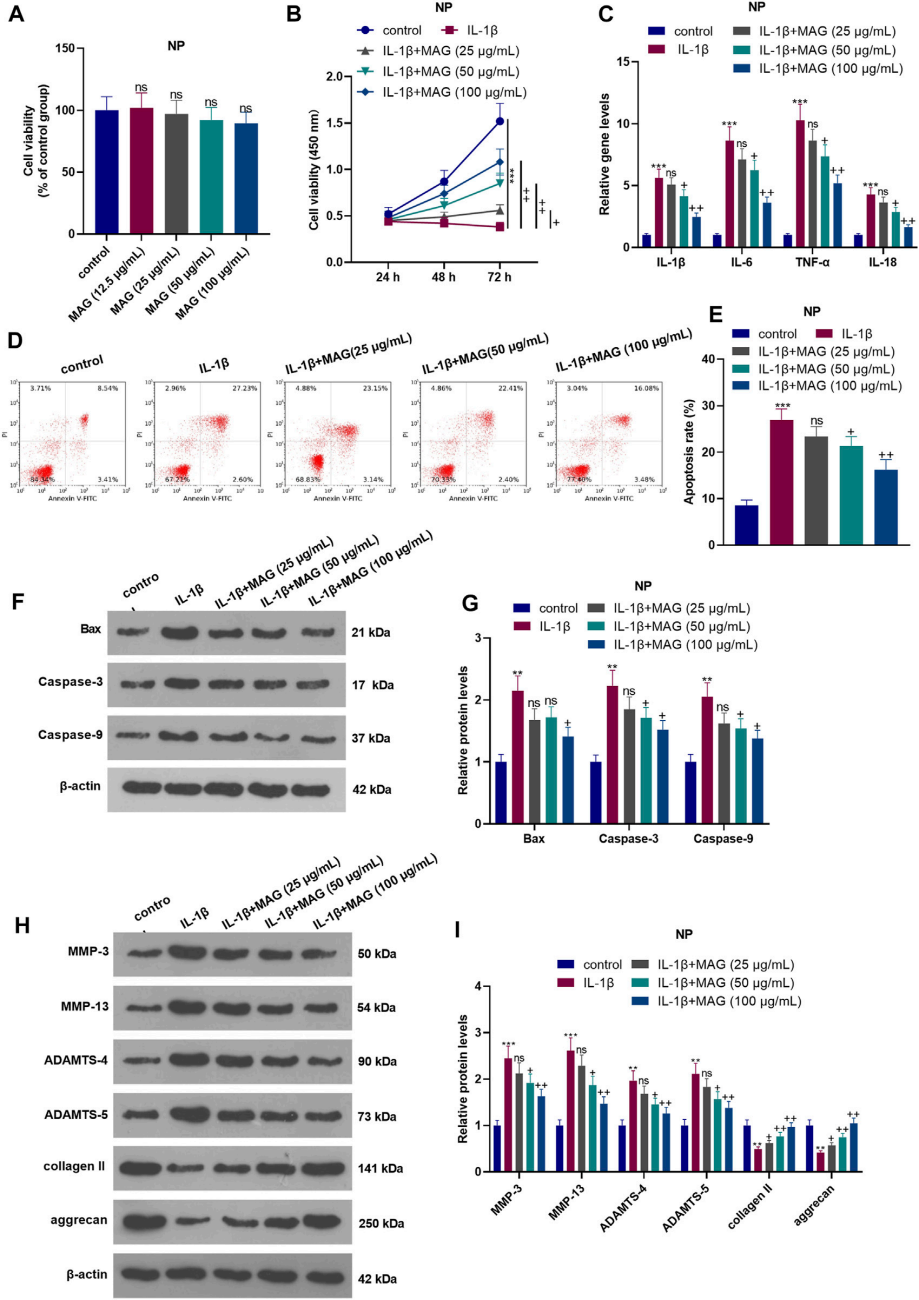
Influences of MAG on IL-1β-mediated NP cell damage. NP cells were dealt with different doses of MAG (12.5, 25, 50, and 100 μg/ml) for 24 h. **(A)**. CCK8 assay was performed to test the viability of NP cells. After IL-1β (20 ng/ml) treatment for 24 h, NP cells were treated with different concentrations of MAG (25, 50, and 100 μg/ml) for 24 h. **(B)**. The CCK-8 assay was applied to test NP cell proliferation at different time points (24, 48, and 72 h). **(C)**. qRT-PCR examined the expression of inflammatory cytokines (IL-1β, IL-6, TNF-α, and IL-18) in NP cells. **(D-E)**. Cell apoptosis was monitored by FCM. **(F-I)**. WB was conducted to determine the expression of Bax, Caspase3, Caspase9, MMP-3, MMP-13, ADAMTS-4, and ADAMTS-5 in NP cells. *n* = 3. ANOVA *p* < 0.01. ns *p* > 0.05, **p < 0.01, ***p < 0.001 (vs control group). ns *p* > 0.05, +*p* < 0.05, ++*p* < 0.01 (vs IL-1β group).

Different doses of MAG (12.5, 25, 50, and 100 μg/ml) were used for treating the NP cells for 24 h. The result of CCK8 suggested that MAG had no significant effect on changing the cell viability of NP cells (compared with control group, *p* > 0.05, Figure 2A). After 24 h of IL-1β treatment, NP cells were treated with different concentrations (25, 50, and 100 μg/ml) of MAG for 24 h. The CCK-8 assay was conducted to examine NP cell viability. As a result, IL-1β abated NP cell viability. Besides, compared with IL-1β treatment alone, MAG treatment (50, 100 μg/ml) based on IL-1β intervention increased cell viability (*p* < 0.05, Figure 2B). After 24 h of IL-1β treatment, NP cells were treated with different concentrations (50, 100 μg/ml) of MAG for 24 h qRT-PCR demonstrated that compared with the control group, IL-1β elevated the expression of IL-1β, IL-6, TNF-α, and 1L-18, while MAG decreased inflammation after IL-1β treatment (*p* < 0.05, Figure 2C). Additionally, FCM demonstrated that the apoptosis rate of NP cells was strengthened after IL-1β treatment, while it was repressed by MAG (vs the IL-1β group) (*p* < 0.05, Figures 2D,E). WB was implemented to determine the expression of apoptosis-related proteins. As exhibited in Figures 2F,G, IL-1β enhanced the expression of apoptosis-related proteins (Bax, Caspase3, Caspase9), but MAG inhibited their expression (vs the IL-1β group) (*p* < 0.05). As shown in Figures 2H,I, the expression of catabolic enzymes (including MMP-3, MMP-13, ADAMTS-4, and ADAMTS-5) were increased, while the expression of ECM compositions (including udigncollagen II and aggrecan) were decreased in NP cells after IL-1β treatment. However, their expression in IL-1β-treated NP cells was partially reversed when MAG was added (*p* < 0.05, Figures 2F–I). These findings indicated that MAG had an inhibitory effect on IL-1β-mediated NP cell damage.

### Impacts of MAG on NP Cell Damage Mediated by “M1” Polarized Macrophage Activation

**FIGURE 3:**
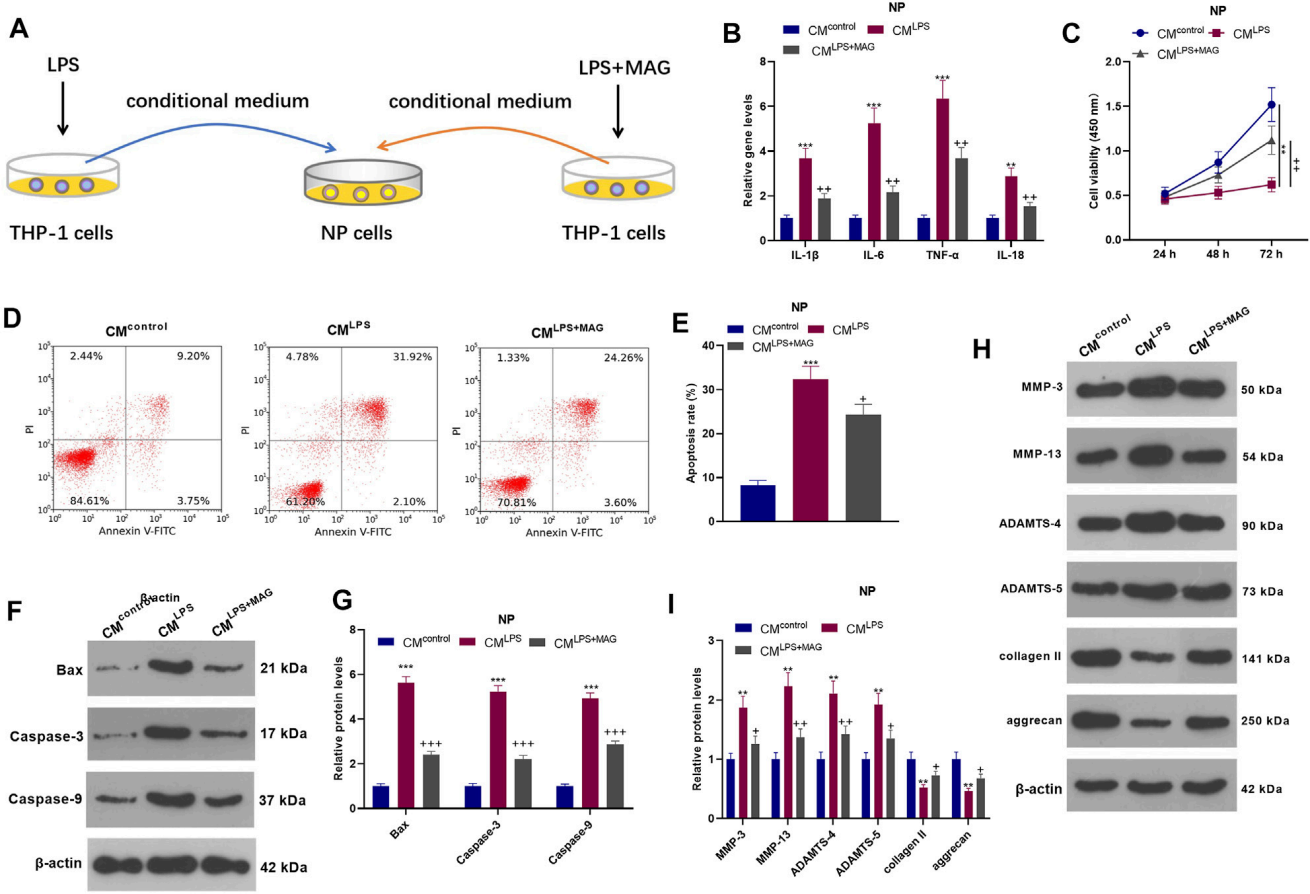
Impacts of MAG on NP cell damage mediated by “M1” polarized macrophage activation. **(A)**.THP1 cells were treated with LPS and/or MAG (100 μg/ml). The condition medium (CM) of THP1 cells were collected and used for treating NP cells for 24 h. **(B)**. qRT-PCR was employed to measure the expression of inflammatory factors (including IL-1β, IL-6, TNF-α, and IL-18) in NP cells. **(C)**. The CCK-8 assay was conducted to test cell viability of NP cells. **(D-E)**. Cell apoptosis of NP cells was monitored by FCM. **(F-I)**. The expression of apoptosis-related proteins and catabolic enzymes was determined by WB. n = 3. ANOVA *p* < 0.01. **p < 0.01, ***p < 0.001 (vs control group). +*p* < 0.05, ++*p* < 0.01, +++*p* < 0.001 (vs CM^LPS^ group).

To explore the interaction between NP cell and THP1 cell, we treated THP1 cells with LPS an/or MAG, then the culture medium of THP1 cells were added into NP cells. QRT-PCR results showed that CM^LPS^ increased the expression of IL-1β, IL-6, TNF-α, and IL-18 in NP cells, while MAG obviously decreased their expression compared with that of the CM group (vs CM^LPS^ group, *p* < 0.05, Figure 3B). The CCK-8 assay confirmed that NP cell viability was decreased after CM^LPS^ treatment However, MAG enhanced NP cell proliferation (vs the CM intervention alone) (vs CM^LPS^ group, *p* < 0.05, Figure 3C). FCM exhibited that CM promoted NP cell apoptosis, while MAG treatment in THP1 cells repressed NP cell apoptosis (vs CM^LPS^ group, *p* < 0.05, Figures 3D,E). WB results showed that CM^LPS^ facilitated the expression of Bax, Caspase3, Caspase9, MMP-3, MMP-13, ADAMTS-4, and ADAMTS-5 and suppressed the expression of collagen II and aggrecan in NP cells. However, the effect of CM^LPS^ on NP cells was partially reversed by treating the THP1 cells with MAG (vs CM^LPS^ group, *p* < 0.05, Figures 3F–I). These findings illustrated that the condition medium from “M1” polarized THP-1 cells damaged NP cells, while MAG partially reversed this damage.

**Table:** 

Gene	Primer sequence (5^’^→3^’^)
IL-1β	F: CTCAACTGTGAAATGCCACC
	R: GAGTGATACTGCCTGCCTGA
IL-6	F: TGTATGAACAACGATGATGCAC
	R: CTGGCTTTGTCTTTCTTGTT
TNF-α	F: CAGGCGGTGCCTATGTCTCA
	R: GCTCCTCCACTTGGTGGTTT
IL-18	F: GCTCACCACAACCTCTACCT
	R: TTCAAGACCAGCCTGACCAA
β-actin	F: CTGTGCCCATCTACGAGGGCTAT
	R: TTTGATGTCACGCACGATTTCC

### MAG Inactivated the HMGB1-MyD88-NF-κB Pathway and NLRP3 Inflammasome in NP Cells

**FIGURE 4:**
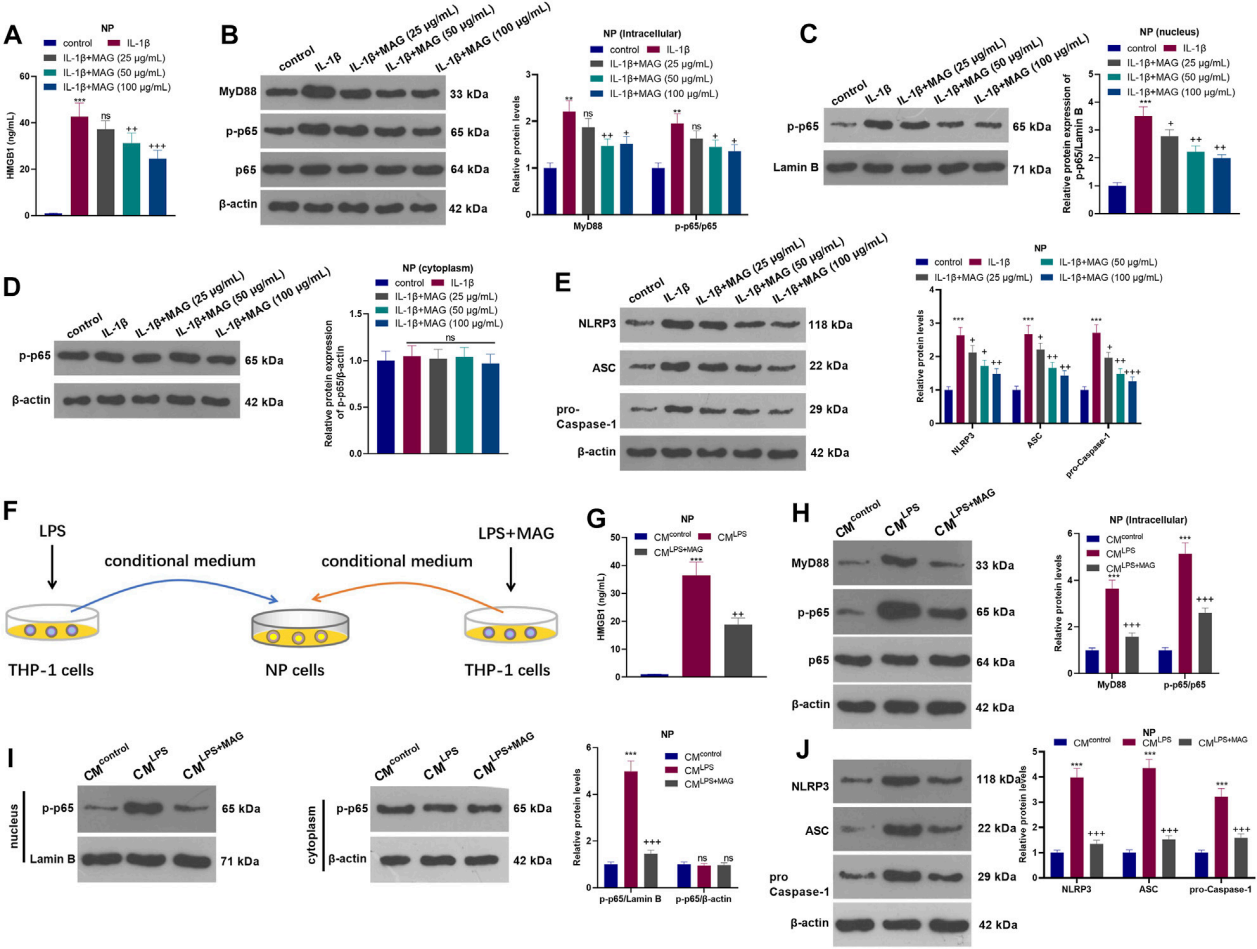
MAG inactivated the HMGB1-MyD88-NF-κB pathway and NLRP3 inflammasome in NP cells. After IL-1β (20 ng/ml) treatment for 24 h, NP cells were treated with different concentrations of MAG (50, 100 μg/ml) for 24 h. **(A)**. The HMGB1 content in the supernatant of NP cells was determined by ELISA. **(B-E)**. WB tested MyD88-NF-κB and NLRP3 expression in NP cells. **p < 0.01, ***p < 0.001 (vs control group). ns*P* >0.05,+*p* < 0.05, ++*p* < 0.01, +++*p* < 0.0001 (vs IL-1β group). **(F)**. THP1 cells were treated with LPS and/or MAG (100 μg/ml). The condition medium (CM) of THP1 cells were collected and used for treating NP cells for 24 h. **(G)**. The HMGB1 content in the supernatant of NP cells was determined by ELISA. **(H-J)**. WB tested MyD88-NF-κB and NLRP3 expression in NP cells. n = 3. ANOVA *p* < 0.01. ns *p* > 0.05, **p < 0.01, ***p < 0.001 (vs CM^control^ group). ns *p* > 0.05, +*p* < 0.05, ++*p* < 0.01 (vs CM^IL−1β^ group).

NP cells were treated with varying concentrations (25, 50, and 100 µg/ml) of MAG after IL-1β treatment. The HMGB1 content was monitored by ELISA. It turned out that IL-1β facilitated the HMGB1 production, while MAG suppressed the HMGB1 production compared with that of IL-1β treatment alone (*p* < 0.05, Figure 4A). WB showed that the profiles of MyD88, p-NF-κB p65 (in the whole cell and nucleus) and NLRP3 in NP cells were elevated after IL-1β treatment, but the elevation was reversed by MAG (*p* < 0.05, Figures 4B–E). On the other hand, the NP cells were dealt with the culture medium from THP1 cells (CM^control^, CM^LPS^, CM^LPS+ MAG^) (Figure 4F). ELISA results manifested that CM^LPS^ strengthened the HMGB1 production in NP cells (vs the CM^control^ group), whereas MAG dampened the HMGB1 production (vs the CM^LPS^ group) (*p* < 0.05, Figure 4G). WB was implemented to monitor the expression of MyD88, NF-κB (in nucleus and cytoplasm) and NLRP3 inflammasome at protein level. The results testified that CM^LPS^ promoted the expression of MyD88, p-NF-κB p65 in the whole NP cells, nucleus translocation of p-NF-κB p65, and NLRP3-ASC-Caspase1 inflammasome, but their expressions were repressed by treating the THP1 cells with MAG (vs CM^LPS^ group, *p* < 0.05, Figures 4H–J). Overall, these results confirmed that MAG inactivated the CM-mediated HMGB1-MyD88-NF-κB pathway and NLRP3 inflammasome in IL-1β-mediated or “M1” polarized THP-1 cells in NP cells.

### IL-1β-Mediated NP Cells Activated “M1” Polarized THP-1 Macrophage

**FIGURE 5:**
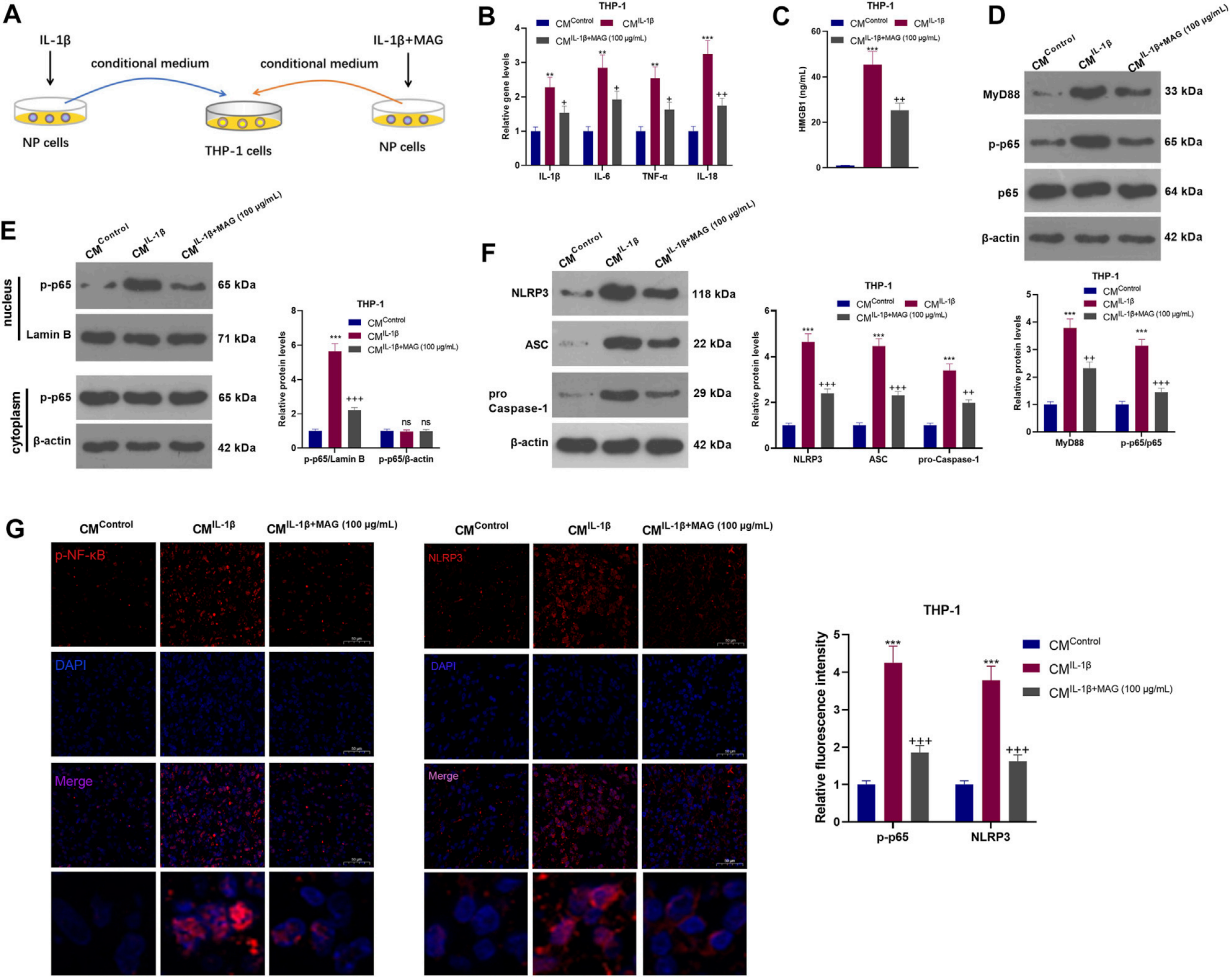
IL-1β-mediated NP cells activated “M1” polarized THP-1 macrophages. **(A)**.After being treated with IL-1β (20 ng/ml), NP cells were treated with a high concentration (100 μg/ml) of MAG. Then, THP-1 cells were treated with the CM of NP cells (CM^Control^, CM^IL−1β^, CM^IL−1β+MAG(100 μg/ml)^) for 24 h. **(B)**. QRT-PCR was implemented to verify the expression of inflammatory cytokines (including IL-1β, IL-6, TNF-α, and IL-18) in THP-1 cells. **(C)**. ELISA was employed to determine the HMGB1 profile in the in the supernatant of THP1 cells. **(D-F)**. The expression of MyD88-NF-κB and NLRP3-ASC-Caspase1 inflammasome was measured by WB. **(G-H)**. Cell immunofluorescence was performed to detect p-NF-κB and NLRP3 in the TBP1 cells (×200). n = 3. ANOVA *p* < 0.01. **p < 0.01, ***p < 0.001 (vs CM^Control^ group). +*p* < 0.05, ++ *p* < 0.01 (vs CM^IL−1β^ group).

NP cells were treated with IL-1β with/without MAG, then the condition medium of NP cells was used for treating THP1 cells (Figure 5A). qRT-PCR results exhibited that CM^IL−1β^ promoted the expression of IL-1β, IL-6, TNF-α, and IL-18 in THP-1 cells (vs the CM^Control^ group), while CM^IL−1β+MAG(100 μg/ml)^ repressed the levels of these inflammatory cytokines (vs the CM^IL−1β^ group) (*p* < 0.05, Figure 5B). ELISA results illustrated that CM^IL−1β^ elevated the HMGB1 profile (vs the CM^Control^ group). However, compared with the CM^IL−1β^ group, CM^IL−1β+MAG(100 μg/ml)^ abated the HMGB1 production (*p* < 0.05, Figure 5C). The expression of the MyD88-NF-κB pathway and NLRP3 inflammasome was monitored by WB and cell immunofluorescence. The results turned out that MyD88, p-NF-κB (both in the whole and nucleus) and NLRP3 were up-regulated after CM^IL−1β^ treatment. However, their expression was reduced by CM^IL−1β+MAG (100 μg/ml)^ (vs the CM^Control^ group) (*p* < 0.05, Figures 5D–G). These findings suggested that IL-1β-mediated NP cells strengthened the activation of “M1” polarized THP-1 macrophages.

### Inhibiting HMGB1 in NP Cells Weakened the NP Cell-Mediated Activation of “M1” Polarized THP-1 Macrophages

**FIGURE 6:**
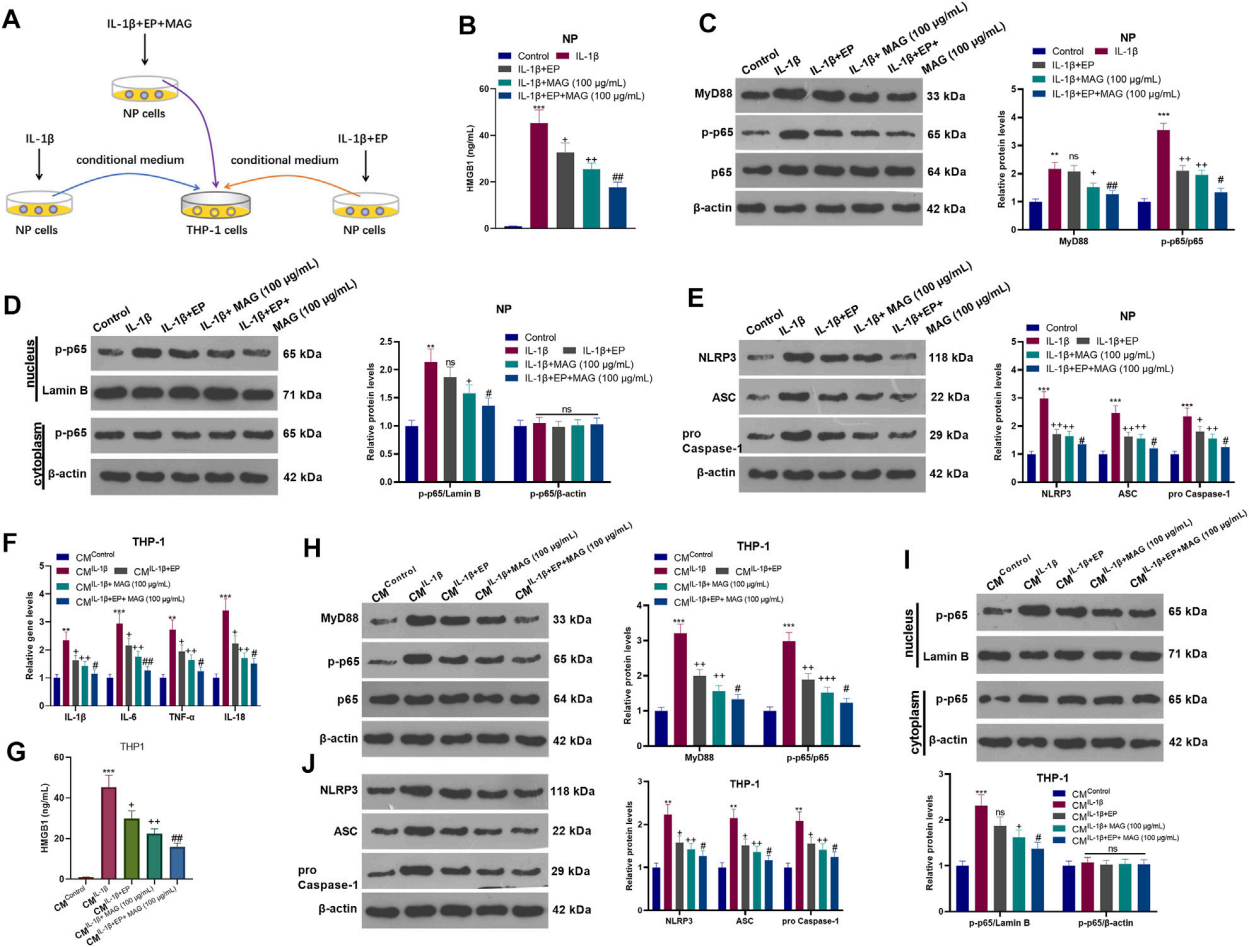
Inhibiting HMGB1 in NP cells weakened NP-mediated “M1” polarized THP-1 macrophage activation. **(A)**.After IL-1β (20 ng/ml) treatment, NP cells were treated with EP (10 mM) and high concentration (100 μg/ml) of MAG for 24 h, respectively. Then, THP-1 cells were treated with the CM of NP cells for 24 h. **(B)**. ELISA was employed to determine the HMGB1 profile in the in the supernatant of NP cells. **(C-E)**. The expression of MyD88-NF-κB and NLRP3-ASC-Caspase1 inflammasome in NP cells was measured by WB. **(F)**. The expression of inflammatory cytokines in THP-1 cells was compared by qRT-PCR. **(G)**. ELISA was employed to determine the HMGB1 profile in the in the supernatant of THP1 cells. **(H-J)**. The expression of MyD88-NF-κB and NLRP3-ASC-Caspase1 inflammasome was measured by WB. *n* = 3. ANOVA *p* < 0.01. **p < 0.01, ***p < 0.001 (vs control/CM^Control^ group). +*p* < 0.05, ++*p* < 0.01 (vs IL-1β/CM^IL−1β^ group). #*p* < 0.05, ##*p* < 0.01 (vs (IL-1β+EP)/CM^IL−1β+EP^ group).

After IL-1β treatment, NP cells were treated with EP and a concentration (100 μg/ml) of MAG. Then, THP-1 cells were treated with CM of NP cells (CM^Control^, CM^IL−1β^, CM^IL−1β+EP^, and CM^IL−1β+EP+MAG^) for 24 h (Figure 6A). ELISA showed that EP declined the HMGB1 level compared with that of the IL-1β group, and MAG intervention on the basis of EP treatment further decreased the HMGB1 expression (vs the IL-1β+EP group) (*p* < 0.05, Figure 6B). WB results illustrated that the expression of MyD88, p-NF-κB p65 (in the whole cell and nucleus) and NLRP3 was reduced after EP or MAG treatment (vs the IL-1β group), while the expression of these proteins was further weakened after MAG treatment on the basis of EP treatment (vs IL-1β+EP group, *p* < 0.05, Figures 6C–E). qRT-PCR revealed that EP repressed the expression of IL-1β, IL-6, TNF-α, and IL-18 in THP1 cells compared with that of the CM^IL−1β^ group, and the expression of these inflammatory cytokines was decreased after MAG treatment based on EP treatment (vs the CM^IL−1β+EP^, *p* < 0.05, Figure 6F). ELISA confirmed that the HMGB1 expression in THP-1 cells was lower than that of the CM^IL−1β^ group after CM^IL−1β+EP^ treatment, and the production of HMGB1 was impeded by high concentrations of MAG (vs the CM^IL−1β+EP^ group) (*p* < 0.05, Figure 6G). The MyD88-NF-κB pathway in THP1 cells were detected. As shown in Figures 6H–J, CM^IL−1β+EP^ repressed the expression of MyD88, p-NF-κB p65 (in the whole cell and nucleus) and NLRP3-ASC-Caspase1 inflammasome in THP-1 cells, and CM^IL−1β+Ep+MAG^ further declined the expression of these proteins (vs the CM^IL−1β+EP^ group, *p* < 0.05, Figures 6H–J). These results testified that inhibiting HMGB1 in NP cells weakened the NP cell-mediated “M1” polarized THP-1 macrophage activation.

## Discussion

It has been reported that IDD is accompanied by an increase in the expression of inflammatory factors, indicating that the occurrence and development of IDD are closely related to inflammation (Navone et al., 2017). The antifungal effect of MAG has been demonstrated (Kim et al., 2018). Additionally, MAG has anti-tumor activity. For example, MAG strengthens the sensitivity of breast cancer cells to doxorubicin, which represses cell proliferation and metastasis and induces cell apoptosis and autophagy (Wei et al., 2020). In the present study, we found that MAG relevels the NP cells damaged caused by activated macrophages via controlling the inflammatory responses both in NP cells and THP1 cells.

IDD is associated with reduced cell proliferation, impaired self-repair, increased inflammation, and catabolism (Wang et al., 2016). NP cells were damaged under the stimulation of inflammatory mediators, including IL-1β. Therefore, IL-1β was used to construct an in-vitro model of IDD on NP cells (Zhang et al., 2019). Our study showed that cell proliferation was decreased, cell apoptosis was increased, the expression of inflammatory factors and catabolic enzymes was facilitated, and ECM enzymes were impeded after IL-1β treatment. These findings suggested that IL-1β damaged NP cells and promoted cell catabolism. Notably, the treatment of MAG reversed these damages on NP cells. Thus, we conclude that MAG has an inhibitory effect on IL-1β-mediated NP cell damage.

To understand the relationship between NP cells and immune regulation, we introduce THP-1 cells (Qin, 2012; Chanput et al., 2014), a mononuclear cell line that can differentiate into various types of macrophages *in vitro* (Chanput et al., 2015). A review of previous studies has found that THP-1 macrophages can get polarized into “M1” state with LPS treatment, thereby regulating inflammation (Chanput et al., 2010; Chanput et al., 2013). Here, THP-1 cells were treated with 100 ng/ml LPS, and the results showed that LPS enhanced the expression of pro-inflammatory cytokines and HMGB1 and activated the MyD88-NF-κB pathway and NLRP3 inflammasome. These indicators testified that LPS induced the “M1” polarization of THP-1 cells. To further define the interaction between NP cells and “M1” polarized macrophages, we treated NP cells with a high concentration (100 μg/ml) of MAG on the basis of treatment with CM of “M1” polarized macrophages. As a result, MAG inactivated “M1” polarized macrophage-mediated NP cell damage. Furthermore, we treated "M1″ polarized macrophages with NP cell medium treated by different factors. It was discovered that CM^IL−1β+MAG(100 μg/ml)^ attenuated “M1” polarized THP-1 macrophage activation induced by CM^IL−1β^. These findings are consistent with the anti-inflammatory effects of MAG.

As reported, HMGB1 is a pro-inflammatory mediator. The extracellular release of HMGB1 is induced when cells are stimulated, and HMGB1 is considered an important new target for treating various inflammatory diseases, including brain injury and cancer (Sun and Chao, 2005; Das et al., 2021). Some other studies revealed that Paeonol reduces sepsis by promoting the miR-339-5p expression to repress the HMGB1 and IKK-β-mediated inflammation (Mei et al., 2019). During the process of IDD, HMGB1 is significantly upregulated in the intervertebral disc (IVD) and promotes the expression of inflammatory cytokines such as prostaglandin E2 (PGE2), TNF-α, IL-6, and IL-8 and matrix metalloproteinases (MMPs) (Rajan et al., 2013; Fang and Jiang, 2016). Presently, we discovered that LPS and IL-1β facilitated the release of HMGB1 in THP-1 and NP cells, while MAG exhibited an inhibitory effect of HMGB1 release. Therefore, it believed that MAG restrains THP1-mediated injury of NP cells via repressing HMGB1 from NP cells.

The NLRP3 inflammasome has been found to contribute to the pathological process and aggravates the disease under external stimuli (Haneklaus and O'Neill, 2015). Data show that the neurotransmitter dopamine (DA) can alleviate systemic inflammation by inhibiting the NLRP3 inflammasome (Yan et al., 2015). Recently, accumulating studies have found that inhibiting NLRP3 inflammasome relieves the progression of IDD. For instance, Morin mitigates thioredoxin-interacting protein (TXNIP)/NLRP3/Caspase-1 signaling pathway-mediated pyroptosis in nucleus pulposus cells and ameliorates intervertebral disc degeneration (Zhou et al., 2021). In the current study, we discovered that MAG hampered the expression of the NLRP3-ASC-Caspase1 inflammasome, which suggested that the anti-inflammatory effect of MAG was partially related to the inhibition of NLRP3 inflammasome.

**FIGURE 7:**
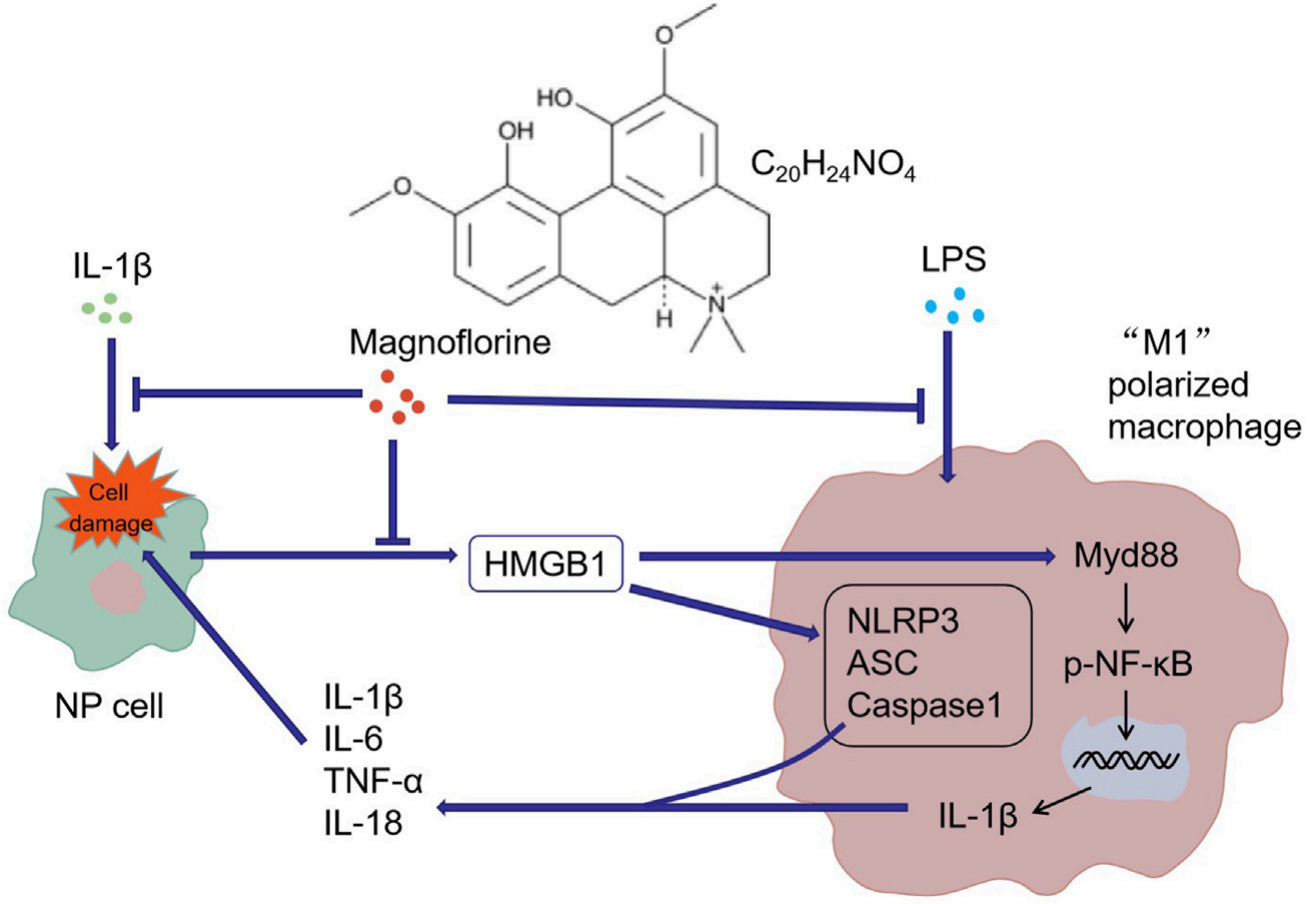
Graphical abstract. IL-1β induced NP cell damage and produced a large amount of HMGB1, while HMGB1 acted on macrophages and induced their “M1” polarization. Similarly, LPS activated the “M1” polarization of macrophages. The "M1″ polarized macrophages produced pro-inflammatory cytokines to induce further damage of NP cells. MAG distinctly dampened the production of HMGB1 in NP cells and the activation of "M1″ polarized macrophages.

Overall, our *in-vitro* study manifested that MAG eased IDD by repressing the inflammatory response. LPS induced the “M1” polarization of THP-1 cells, while “M1” polarized macrophages induced NP cell damage and thus led to IDD. MAG attenuated inflammation by down-regulating the HMGB1/MyD88/NF-κB pathway and NLRP3 inflammasome, thereby alleviating the “M1” polarized macrophage-mediated in-vitro IDD model (Figure 7). However, further *in-vivo* experiments were needed to confirm the protective and anti-inflammatory effects of MAG in IDD.

## Data Availability

The raw data supporting the conclusions of this article will be made available by the authors, without undue reservation.
